# MicroRNA-625-3p improved proliferation and involved chemotherapy resistance via targeting PTEN in high grade ovarian serous carcinoma

**DOI:** 10.1186/s13048-021-00939-1

**Published:** 2022-01-14

**Authors:** Lili Zhong, Xiumin Liu, Lixing Wang, Yu Liu, Duohan Zhang, Yinlong Zhao

**Affiliations:** 1 The Second Hospital of Jilin University, Changchun, 130041 China; 2 Clinical Laboratory, Second Hospital of Jilin University, Changchun, 130041 China; 3 Department of Nuclear Medicine, The Second Hospital of Jilin University, No. 218, Ziqiang Street, Nanguan, Changchun, 130041 Jilin People’s Republic of China

**Keywords:** miR-625-3p, HGSOC, Apoptosis, PTEN

## Abstract

**Objective:**

High-grade serous ovarian cancer (HGSOC) is an aggressive gynaecological malignancy and associated with poor prognosis. Here we examined the effects of miR-625-3p on proliferation, treatment, migration and invasion in HGSOC.

**Methods:**

The proliferation of HGSOC cells was evaluated by MTT assay. Transwell assay was performed to examine migration and matrigel assay were used to assess invasion. The effect of miR-625-3p on cisplatin-induced apoptosis was investigated by Caspase-Glo3/7 assay. The dual-luciferase reporter assay was carried out to confirm the potential binding site.

**Results:**

Overexpression of miR-625-3p promoted proliferation, and increased migration and invasion in HGSOC cells. MiR-625-3p significantly inhibited cisplatin sensitivity in HGSOC cells. Meanwhile, miR-625-3p decreased cisplatin-induced apoptosis by regulation of BAX and Bcl-2 expression. Furthermore, aberrant expression of miR-625-3p changed PTEN expression by directly binding to 3’UTR of PTEN. Further study showed miR-625-3p expression was higher in human HGSOC tissue than normal ovarian tissues and associated with higher clinical stage.

**Conclusions:**

miR-625-3p promotes HGSOC growth, involves chemotherapy resistance and might serve as a potential biomarker to predict chemotherapy response and prognosis in HGSOC.

## Introduction

HGSOC is consisted of 60-80% of ovarian epithelial carcinoma, and patients with HGSOC most often present at advanced clinical stage and have poor outcome [1]. The first-line treatment of HGSOC is debulking surgery followed by chemotherapy [2]. Cisplatin interferes cell division and inhibits cancer cell proliferation, and has been used as a chemotherapy reagent for a variety of cancers including HGSOC [3]. However, studies have shown that cancer cells develop cisplatin resistance by reducing accumulation [4, 5]. Patients who develop cisplatin resistance often have shorter survival time. Therefore, it is critical to find out the molecular mechanisms of cisplatin resistance and develop effective treatments in patients with HGSOC.

Recently, growing evidence has shown that microRNAs impact essential cellular processes such as differentiation, proliferation and apoptosis in organ development and tumorigenesis by directly binding to 3’-untranslated region of target mRNAs [6, 7]. MiRNA profiling studies have shown aberrant miRNA expression in a variety of neoplasms including ovarian cancer [8]. Yang et al. showed that miRNA-802 inhibited growth and migration in ovarian cancer by regulating YWHAZ [9]. Rao et al reported that miR-195-5p suppressed ovarian cancer cell proliferation by directly targeting 3’-UTR of MICU1 mRNA and repressing MICU1 expression [10]. Xing et al found that miR-598 inhibited ovarian cancer cell growth and metastasis by regulating unconventional prefoldin RPB5 interactor, a member of the prefoldin family of molecular chaperones [11]. Interestingly, some studies have demonstrated that circulating miRNAs can be potential diagnostic biomarkers in patients with ovarian cancer [12, 13].

In this study, we evaluated the effects of miR-625-3p in growth, cisplatin sensitivity, migration and invasion using HGSOC cells and tissue.

## Materials and methods

### Ovarian cancer cell lines

Two HGSOC cell lines, OVCAR3 and OVCAR4, were purchased from the American Type Culture Collection (ATCC, USA) and grown in Dulbecco's Modified Eagle's Medium (DMEM) supplemented with 10% fetal bovine serum, 100 units of penicillin/ml and 100 mg of streptomycin/ml (Invitrogen, USA).

### MicroRNA transfection

The miR-625-3p mimic and miR-625-3p inhibitor (Invitrogen, USA) were transfected to ovarian cancer cells using Lipofectamine 2000 (Invitrogen, USA). The scramble microRNA was used as negative control (Invitrogen, USA).

### Quantitative real-time polymerase chain reaction (qRT-PCR)

Total RNA was extracted from ovarian cancer cells using TRIzol reagent (Invitrogen, USA). Quantitative Real-Time PCR (qRT-PCR) was performed to detect miR-625-3p expression level. The reactions were performed as follows: 94°C for 1 min, 36 cycles at 94°C for 30 s, 60°C for 25 s, and a dissociation stage. The primers were synthesized by Sigma (USA) and the sequences were as follow: miR-625-3p forward: 5’- ATTAGATTGCTAACTAGC-3’ and reverse 5’- TTAGTACGAATTATCGTAA-3’. U6 was included as endogenous control.

### Cell proliferation assay

MTT assay was used to evaluate ovarian cancer cell proliferation. Briefly, OVCAR3 and OVCAR4 cells were seeded in 96-well plates and cultured overnight. Then different concentration of cisplatin (0, 2, 4 and 6 μM) was added. Every 48 hours, MTT reagents was added and incubated for 4 hours. Then DMSO was added to dissolve the formed crystal. The signal was detected on a microplate reader (Invitrogen, USA) at 570 nm.

### Caspase 3/7 apoptosis assay

Ovarian cancer cells were incubated with different concentrations of cisplatin (0, 2, 4 and 6 μM) for 48 hours. Then Caspase-Glo reagent (Promega, USA) was added and incubated for 2 hours. Luminescence was evaluated with parameters of 1 minute lag time and 0.5 second/well read time in a plate-reading luminometer (ThermoFisher, USA).

### Transwell assay

The invasion assay was conducted using upper chambers coated with Matrigel (BD Bioscience, USA). The migration assay was performed using the top chambers without Matrigel. Briefly, ovarian cancer cells were placed in upper chambers in serum free culture medium, and culture medium supplemented with 10% FBS was added into lower chambers. After 18 hours culture, the invaded cells on the bottom of membrane were stained and counted at randomly fields.

### Western blotting

Total protein was extracted from ovarian cancer cells and separated by SDS-PAGE gel electrophoresis. The proteins were transferred to PVDF membranes (Sigma, USA). After blocking with 5% nonfat dried milk, the PVDF membranes were incubated with different primary antibodies (Table 1) overnight at 4°C with gentle agitation, followed by incubation with secondary antibodies. The signal on the PVDF membrane was examined by enhanced chemiluminescence reagent (Pierce, USA).

**Table 1:** The antibodies used in western blot

Antibody	Vendor	Dilution
BAX	Cell signaling Technology, USA	1:1000
BCL-2	Santa cruz Biotechnology, USA	1:1000
p-AKT	Cell signaling Technology, USA	1:500
PTEN	Santa cruz Biotechnology, USA	1:500
AKT	Cell signaling Technology, USA	1:1500
GAPDH	Santa cruz Biotechnology, USA	1:2000

### Luciferase assay

The potential binding sites of miR-625-3p were identified by checking miRDB online database. The sequence of PTEN 3'-UTR containing the putative miR-625-3p binding site was amplified from human normal cell genomic DNA. Using the In-Fusion Dry-Down PCR Cloning Kit (Clontech, USA), the amplified sequence was cloned into the Xbal site of the pmirGLO Dual-Luciferase miRNA Target Expression Vector (Promega, USA). Three point mutations were introduced into the seed region of the miR-625-3p binding sites, which was used as control. OVCAR3 cells were co-transfected with Luc-PTEN and miR-625-3p using Lipofectamine RNAiMAX. After 48 hours, luciferase activities were measured using the Dual-Luciferase Reporter Assay system (Promega, USA).

### MiR-625-3p expression on human ovarian cancer tissue

Formalin-fixed paraffin-embedded (FFPE) tissue was collected from 155 patients with ovarian cancer between 2005 and 2020. This study was approved by ethical committee of Jilin University. The informed consents were collected from every patient. The diagnosis was confirmed by two experienced pathologists and qRT-PCR were performed using total RNA extracted from FFPE tissue to evaluate miR-625-3p levels.

### Immunohistochemical analysis

PTEN and Ki-67 immunostains were performed. The normal ovarian tissue adjacent to ovarian cancer tissue was used as control. Immunohistochemical stain was performed on automated stainer (Bond RX, USA) using PTEN (Cell Signaling, 1:1500) and Ki-67 (Cell Signaling, 1:1500) antibodies.

### Statistical analysis

SPSS 12.0 statistical software (IBM Corp, USA) was used for statistical analysis. P < 0.05 was considered statistically significant using one-way analysis of variance (Tukey, ANOVA).

## Results

### MiR-625-3p increased growth of OVCAR3 and OVCAR4 and inhibited cisplatin sensitivity

**Fig. 1:**
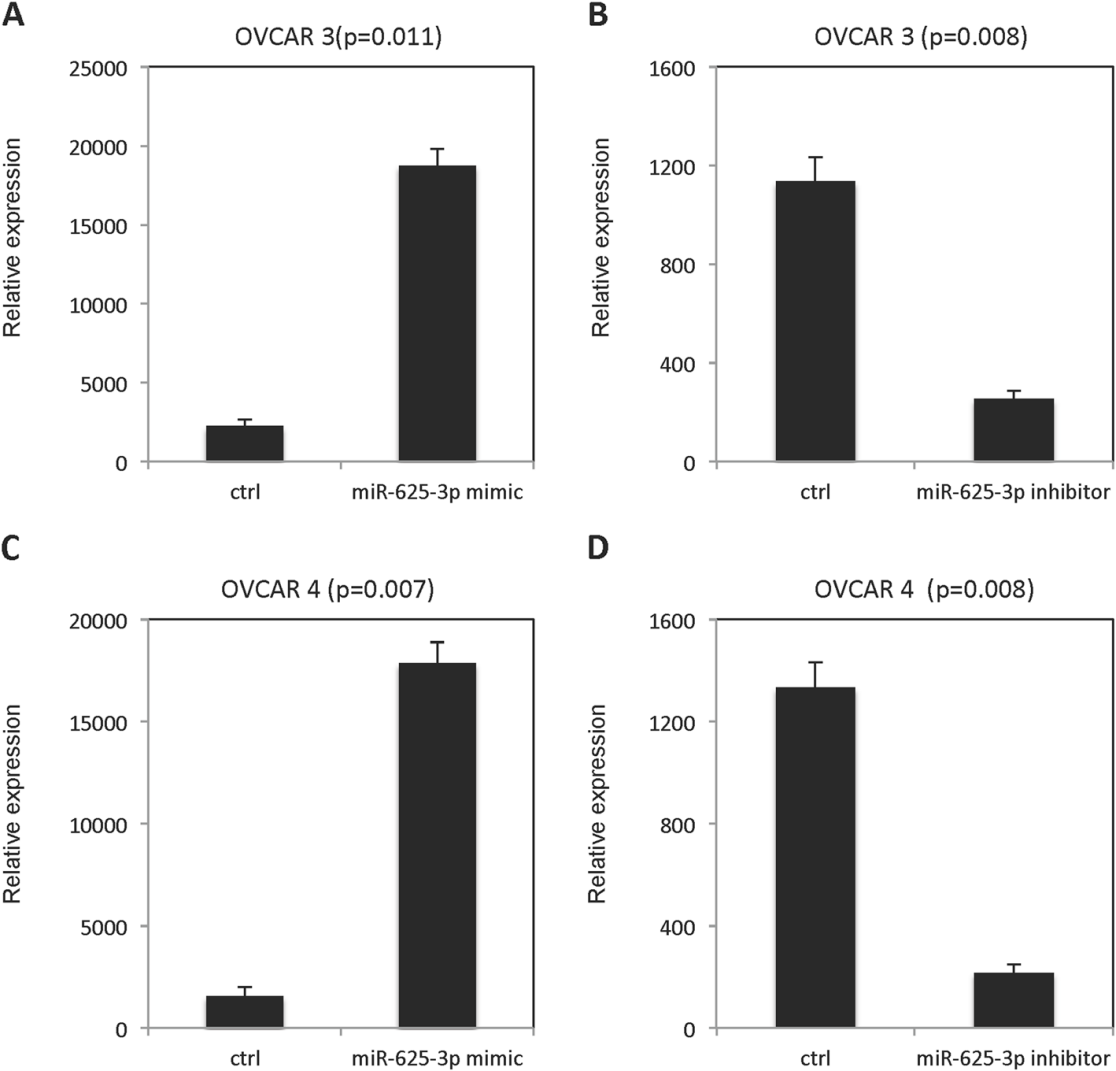
miR-625-3p expression in OVCAR3 and OVCAR4 cell after transfected either with mimic or inhibitor. **A** miR-625-3p expression in OVCAR3 cells transfected with miR-625-3p mimic. **B** miR-625-3p expression in OVCAR3 cells transfected with miR-625-3p inhibitor. **C** miR-625-3p expression in OVCAR4 cells transfected with miR-625-3p mimic. **D** miR-625-3p expression in OVCAR4 cells transfected with miR-625-3p inhibitor

**Fig. 2:**
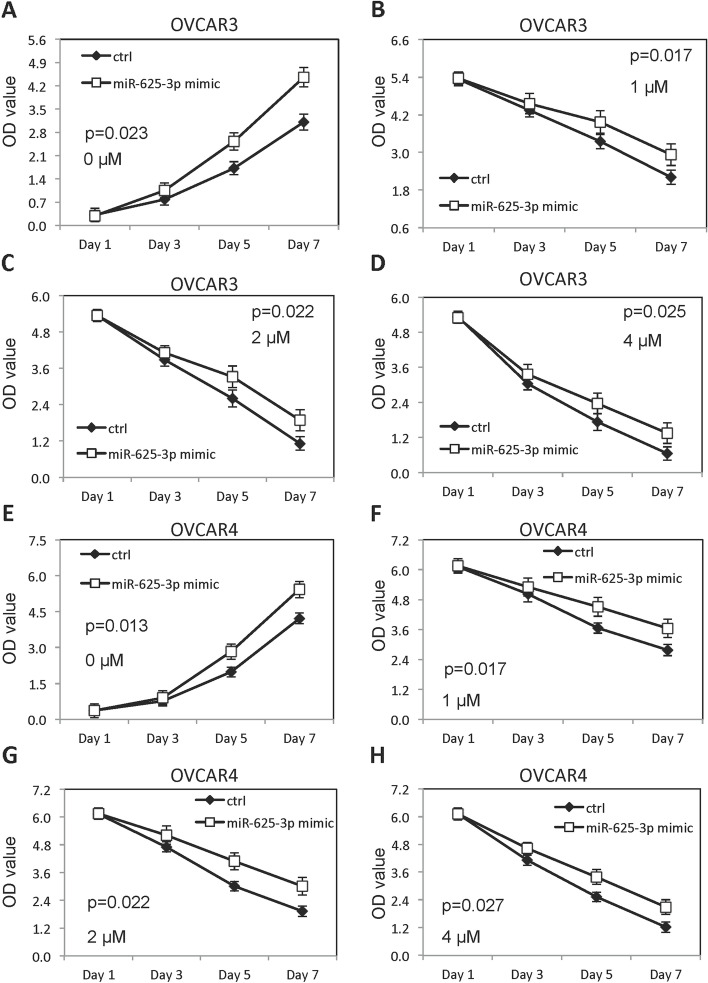
Forced overexpression of miR-625-3p increased growth and inhibited cisplatin sensitivity. **A** Growth of OVCAR3 cells forced overexpressed miR-625-3p without cisplatin. **B-D** Growth of OVCAR3 cells forced overexpressed miR-625-3p in presence of different doses of cisplatin (1, 2 and 4μM). **E** Growth of OVCAR4 cells forced overexpressed miR-625-3p without cisplatin. **F-H** Growth of OVCAR4 cells forced overexpressed miR-625-3p in presence of different doses of cisplatin (1, 2 and 4μM)

**Fig. 3:**
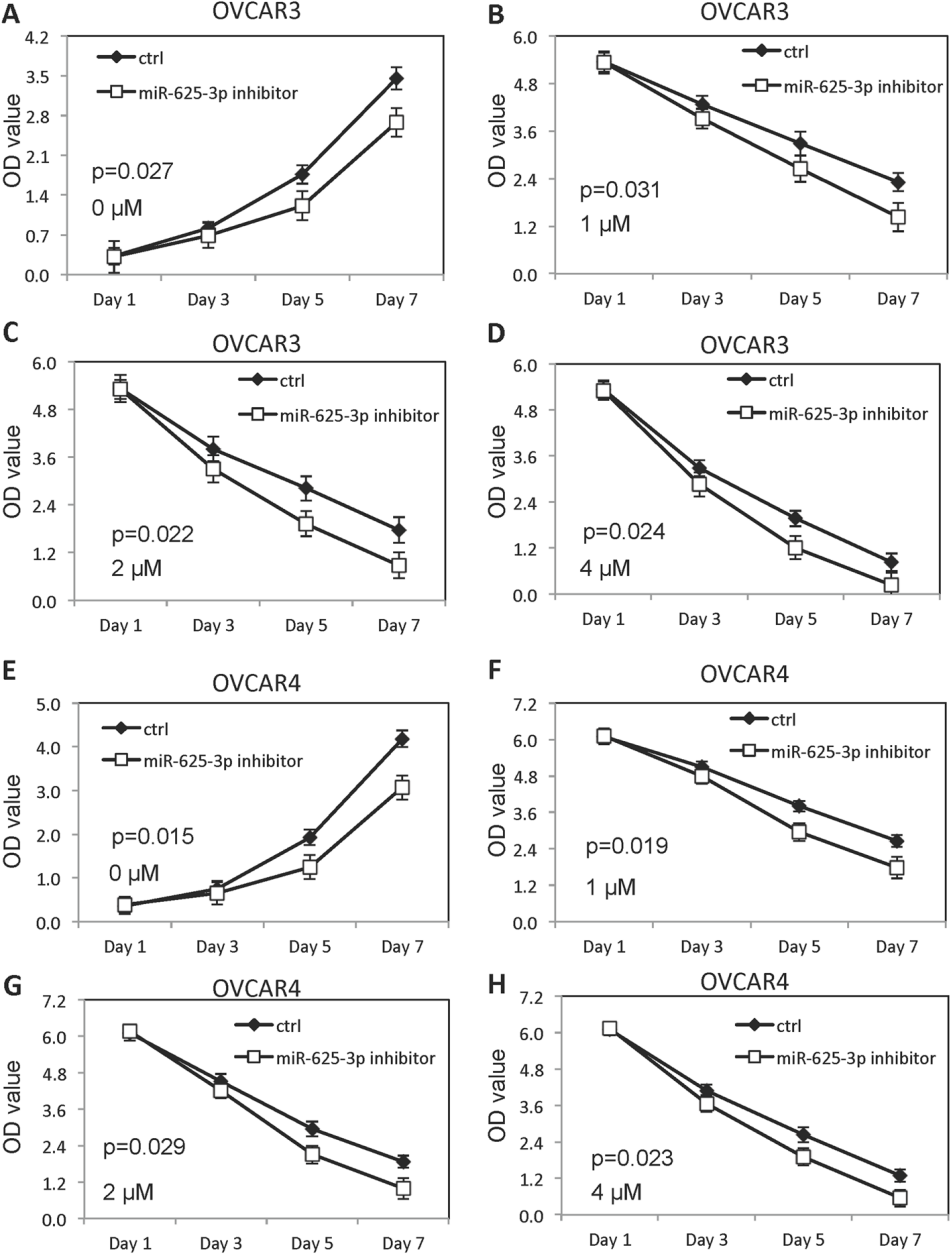
Low level of miR-625-3p decreased growth and promoted cisplatin sensitivity. **A** Growth of OVCAR3 cells decreased miR-625-3p without cisplatin. **B-D** Growth of OVCAR3 cells decreased miR-625-3p in presence of different doses of cisplatin (1, 2 and 4μM). **E** Growth of OVCAR4 cells decreased miR-625-3p without cisplatin. **F-H** Growth of OVCAR4 cells decreased miR-625-3p in presence of different doses of cisplatin (1, 2 and 4μM)

miR-625-3p expression was dramatically increased in OVCAR3 (Fig. 1A) and OVCAR4 (Fig. 1C) cells after transfection of miR-625-3p mimic. However, miR-625-3p expression was significantly decreased in OVCAR3 (Fig. 1B) and OVCAR4 (Fig. 1D) cells after transfection of miR-625-3p inhibitor. Growth of OVCAR3 and OVCAR4 cells in absence and presence of cisplatin was checked by MTT assay. As shown in Fig. 2, forced overexpression of miR-625-3p improved growth of OVCAR3 cells (Fig. 2A) and OVCAR4 cells (Fig. 2E). Meanwhile, forced overexpression of miR-625-3p inhibited cisplatin sensitivity in a dose dependent manner both in OVCAR 3 cells (Fig. 2B-D) and OVCAR4 cells (Fig. 2F-H). On the contrast, low expression of miR-625-3p decreased growth of OVCAR3 cells (Fig. 3A) and OVCAR4 cells (Fig. 3E). Meanwhile, low expression of miR-625-3p improved cisplatin sensitivity in a dose dependent manner both in OVCAR 3 cells (Fig. 3B-D) and OVCAR4 cells (Fig. 3F-H).

### MiR-625-3p decreased cisplatin-induced apoptosis in OVCAR3 and OVCAR4 cells

**Fig. 4:**
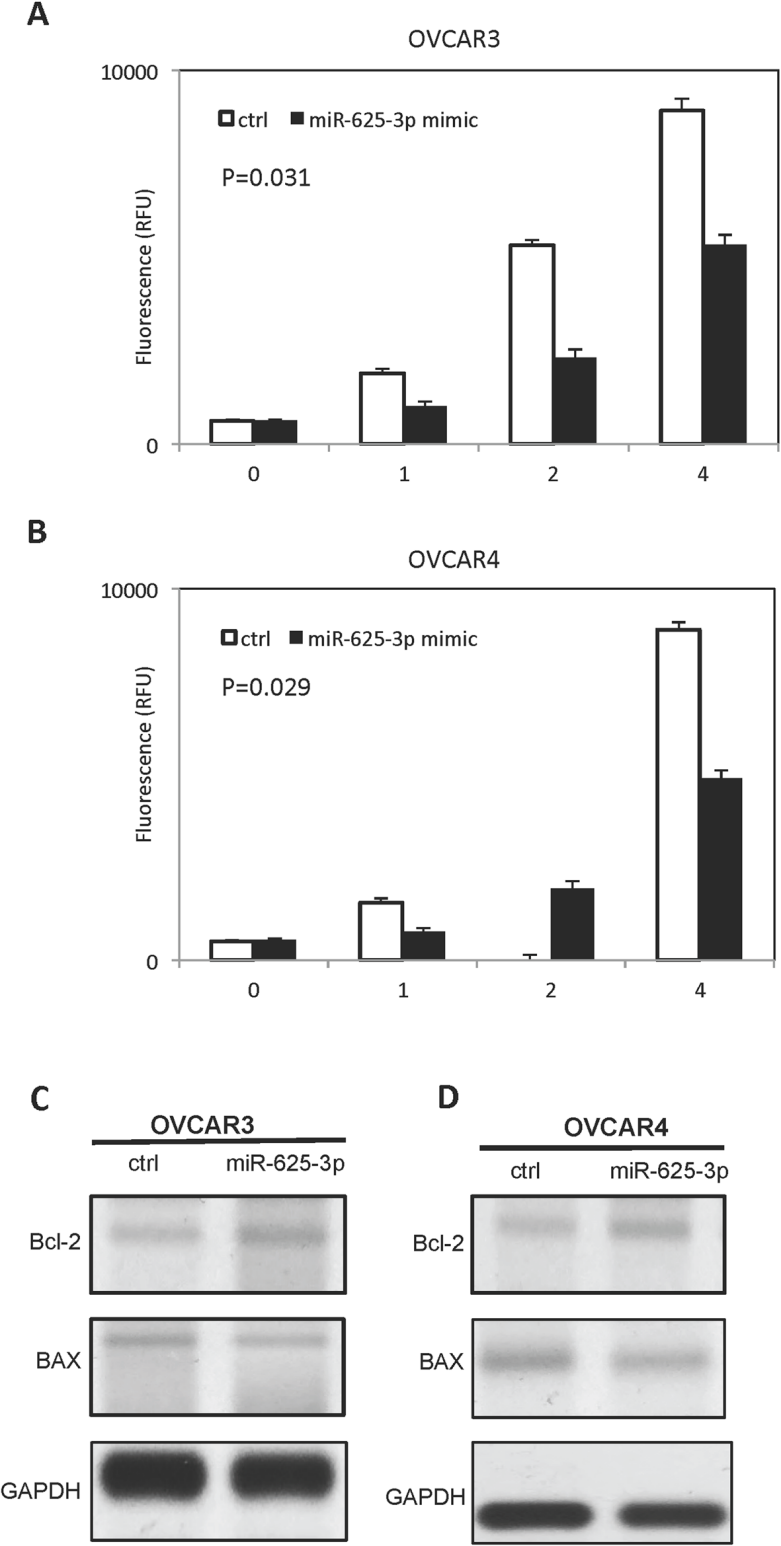
miR-625-3p inhibits cisplatin-induced apoptosis. **A** miR-625-3p decreased caspase 3/7 activity in OVCAR3 cells treated with cisplatin in dose dependent manner. **B** miR-625-3p decreased caspase 3/7 activity in OVCAR4 cells treated with cisplatin in dose dependent manner. **C-D** miR-625-3p regulates BAX and BCL-2 expression in OVCAR3 and OVCAR4 cells treated with cisplatin

Caspase 3/7 activity was performed to examine the effect of miR-625-3p on cisplatin-induced apoptosis. Forced overexpression of miR-625-3p decreased cisplatin-induced apoptosis in OVCAR3 cells (Fig. 4A) and OVCAR4 cells (Fig. 4B). Meanwhile, high level of miR-625-3p inhibited BAX and increased Bcl-2 in OVCAR3 cells and OVCAR4 cells (Fig.4C-D).

### MiR-625-3P promoted invasion and migration in OVCAR3 and OVCAR 4 cell

**Fig. 5:**
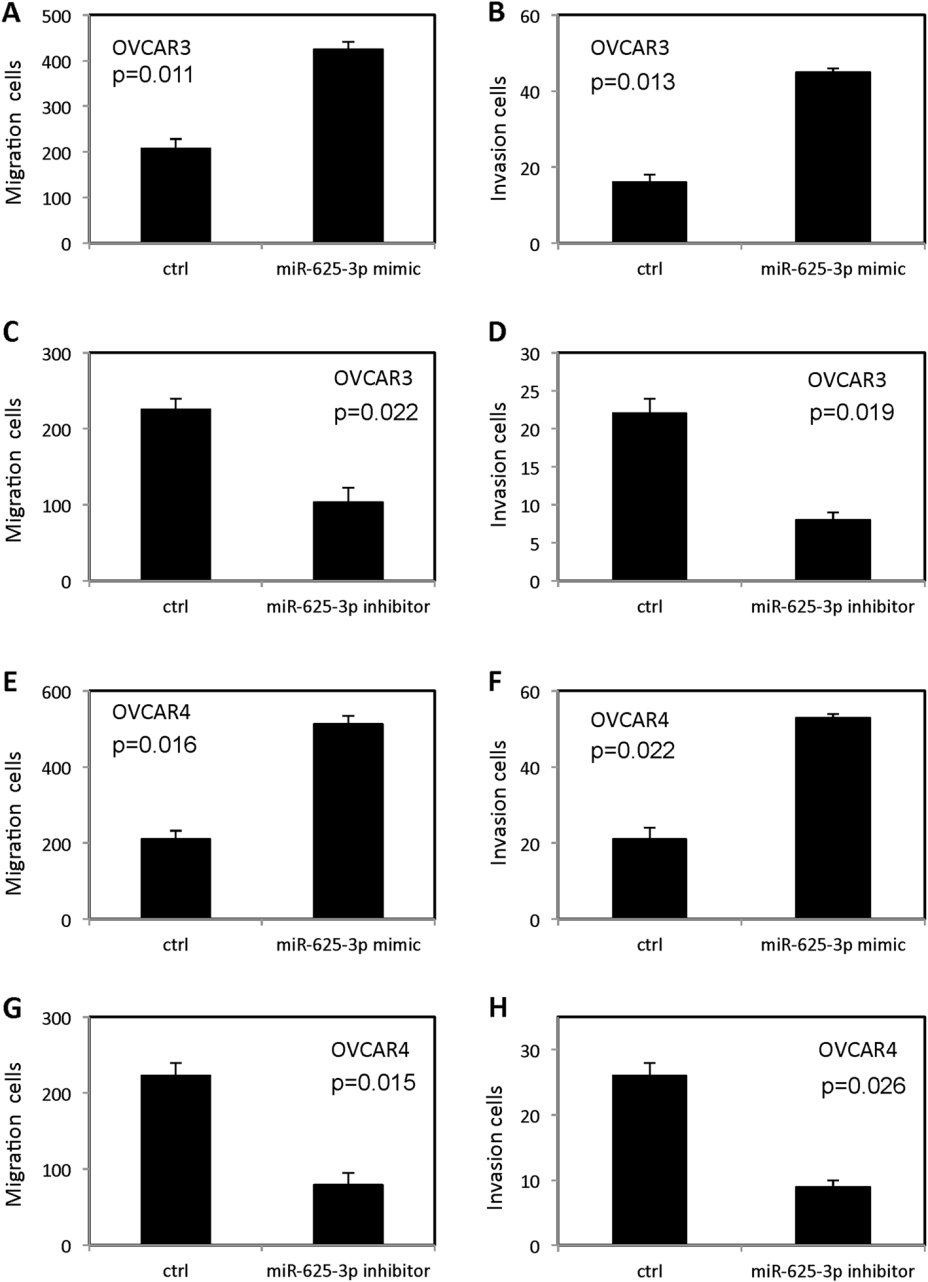
miR-625-3p regulated invasion and migration in OVCAR3 and OVCAR4 cells. **A-B** Forced overexpression of miR-625-3p promoted migration and invasion in OVCAR3 cells. **C-D** Low level miR-625-3p inhibited migration and invasion in OVCAR3 cells. **E-F** Forced overexpression of miR-625-3p promoted migration and invasion in OVCAR4 cells. **G-H** Low level miR-625-3p inhibited migration and invasion in OVCAR4 cells

As shown in Fig. 5, forced overexpression of miR-625-3p promoted migration (Fig. 5A and E) and invasion (Fig. 5B and F) in OVCAR3 and OVCAR4 cells. In contrast, low expression of miR-625-3p inhibited migration (Fig. 5C and G) and invasion (Fig. 5D and H) in OVCAR3 cells and OVCAR4 cells.

### MiR-625-3p decreased PTEN expression in OVCAR3 and OVCAR4 cells by directly binding to 3’-UTR of PTEN

**Fig. 6:**
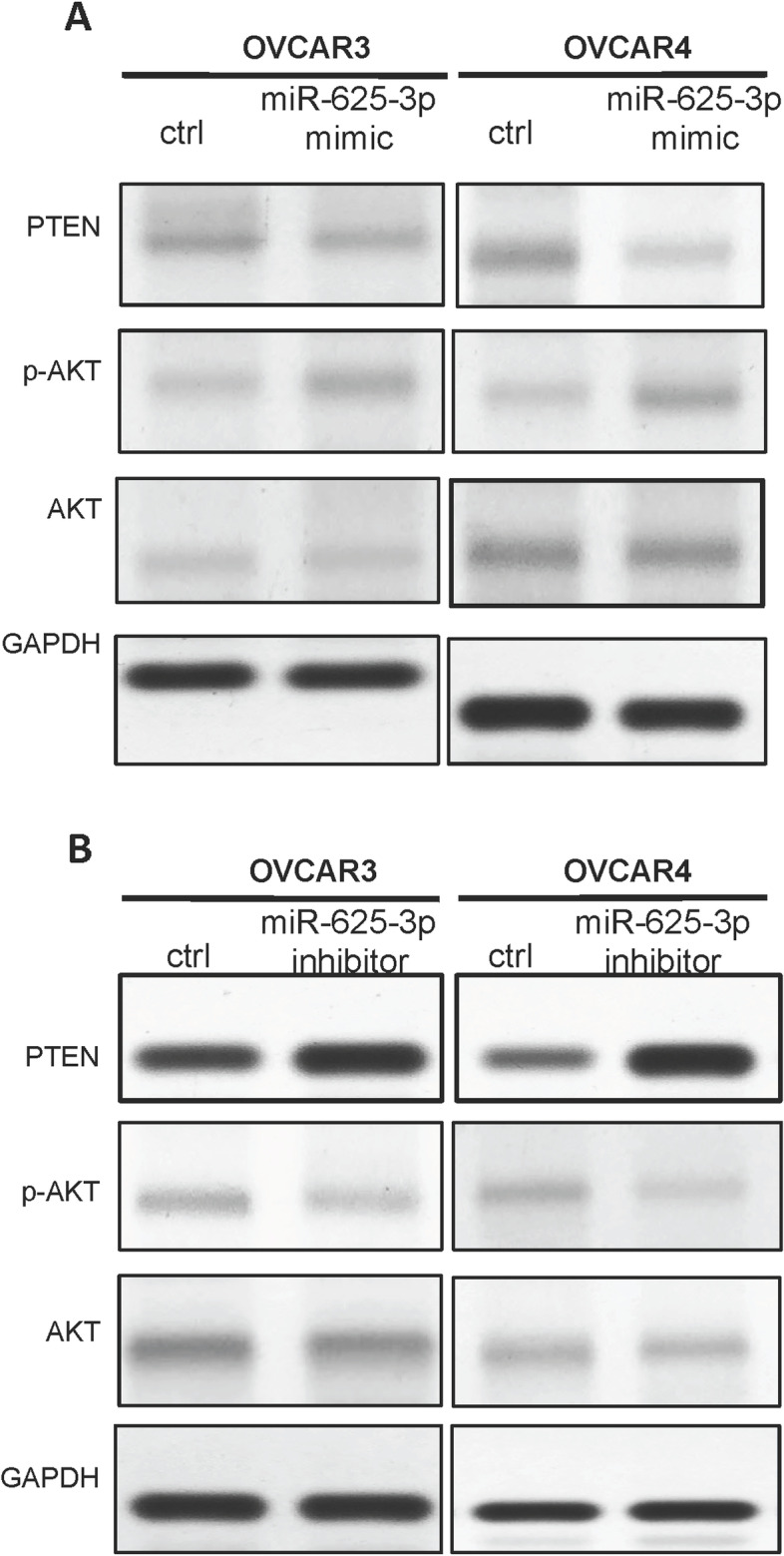
miR-625-3p regulated PTEN/AKT protein expression. **A** Forced overexpression miR-625-3p regulated expression of p-AKT, AKT and PTEN in OVCAR3 and OVCAR4 cells. **B** Low level of miR-625-3p regulated expression of p-AKT, AKT and PTEN in OVCAR3 and OVCAR4 cells

Forced overexpression of miR-625-3p deceased PTEN expression and increased p-AKT expression in OVCAR3 (Fig. 6A) and OVCAR4 (Fig. 6B).

**Fig. 7:**
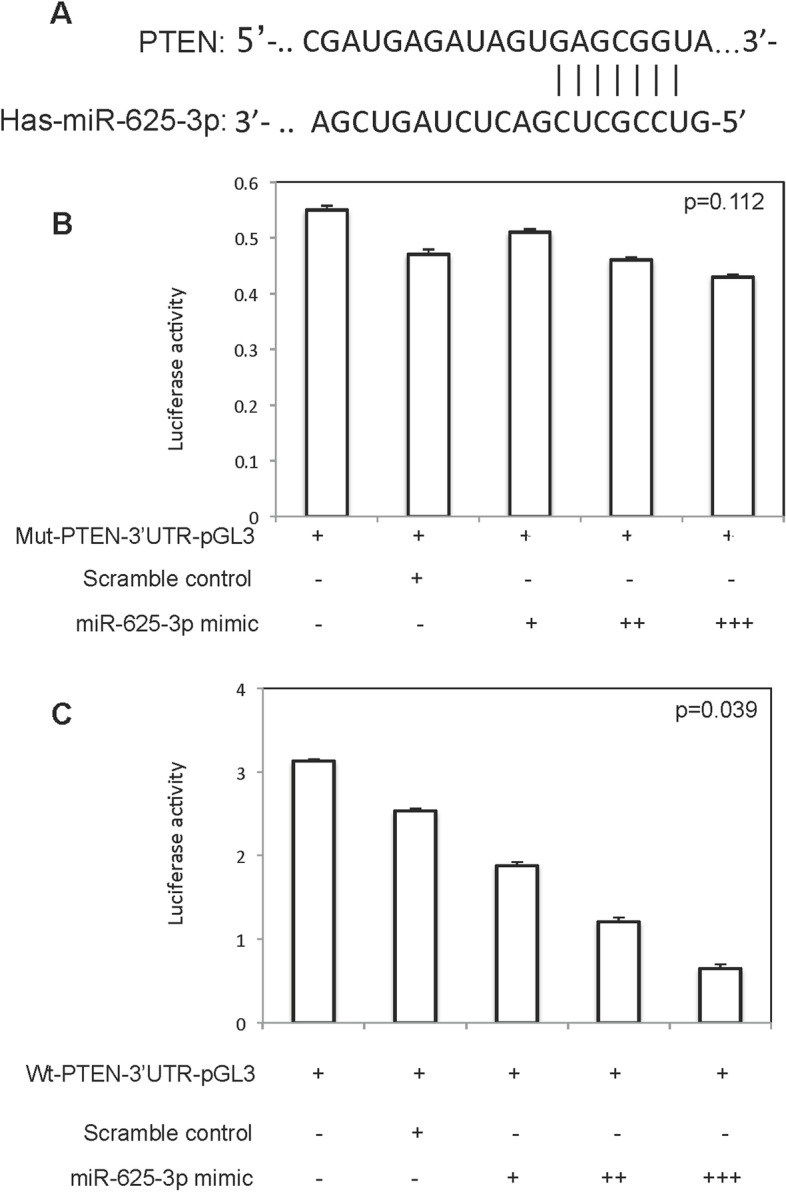
miR-625-3p directly targeted PTEN. **A** The possible binding site of miR-625-3p to PTEN; **B** Luciferase activity of OVCAR3 cells co-transfected miR-625-3p mimic with mut-PTEN-3’-UTR-pGL3; **C** Luciferase activity of OVCAR3 cells co-transfected miR-625-3p mimic with wt-PTEN-3’-UTR-pGL3

The dual-luciferase reporter assay demonstrated that luciferase activity did not change when mu-PTEN-3’-UTR-pGL3 was co-transfected with miR-625-3p, while luciferase activity of Luc-PTEN was decreased when wt- PTEN-3’-UTR-pGL3 was co-transfected with miR-625-3p (Fig. 7).

### MiR-625-3p level on human ovarian high-grade serous cancer tissue

**Fig. 8:**
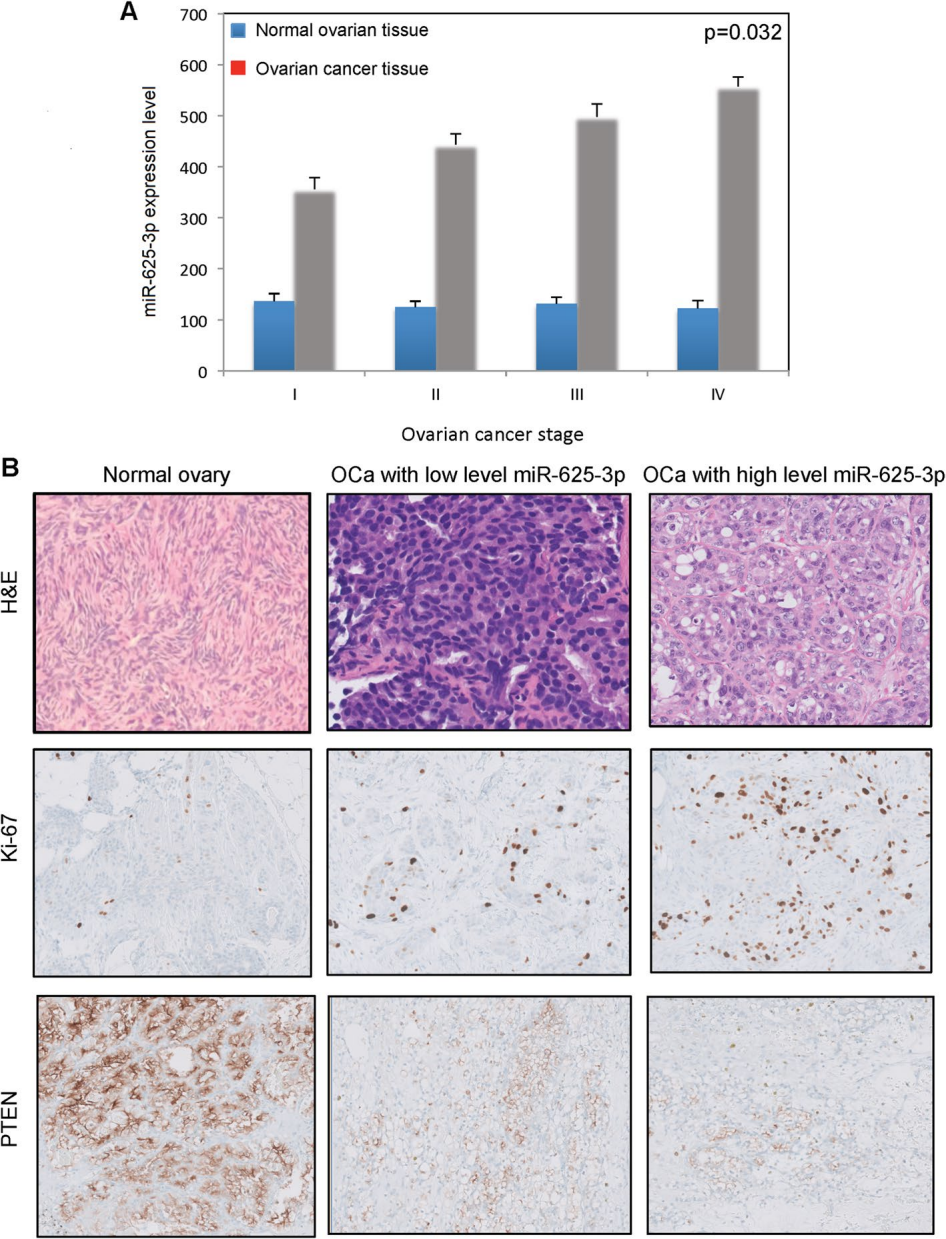
miR-625-3p level in human high-grade serous carcinoma tissue. **A** miR-625-3p level in different stage ovarian cancer. **B** Immunohistochemical studies for Ki-67 and PTEN on normal ovarian tissue, ovarian cancer with low level miR-625-3p and ovarian cancer with high level miR-625-3p

Formalin-fixed paraffin-embedded (FFPE) tissue was obtained and qRT-PCR was carried out to examine miR-625-3p expression. We found that miR-572 expression was correlated with ovarian cancer stage (Table 2, Fig. 8A). In high-grade serous carcinoma expressing high-level miR-625-3p (Fig. 8B), Ki-67 proliferation index is increased. However, PTEN expression was decreased in high-grade serous carcinoma expressing high-level miR-625-3p.

**Table 2:** miR-625-3p expression level and Ki-67 index in different stage high grade ovarian serous cancer

Stage	Number of patients	miR-625-3p levels in normal breast tissue	miR-625-3p levels in invasive cancer tissue	Ki-67 index (per 2 mm2)
IA	25	103±6	221±33	16±2
IB	28	122±11	239±25	17±1
IC	21	110±10	223±29	15±2
IIA	21	101±11	389±21	22±2
IIB	18	93±6	321±25	26±4
IIIA	14	101±9	471±23	35±4
IIIB	12	117±7	464±21	37±2
IIIC	7	106±11	485±26	41±3
IVA	6	102±14	536±17	47±2
IVB	3	131±19	544±19	49±5

## Discussion

Emerging evidence has demonstrated that miR-625-3p plays important roles in variety of different neoplasms. Fang et al showed that miR-625-3p improved growth, migration and invasion in thyroid papillary carcinoma by targeting astrocyte elevated gene 1 [14]. Rasmussen et al reported that expression of miR-625-3p was up-regulated in colorectal cancer cells and associated with resistance to oxaliplatin treatment and reduced overall survival [15]. Krischner and his co-workers found that miR-625-3p concentration is dramatically higher in plasma of patients with malignant pleural mesothelioma and demonstrated miR-625-3p could serve as a potential diagnostic marker [16]. Li et al showed that miR-625-3p decreased lymph node metastasis in gastric carcinoma by directly targeting enhancer of zeste homolog 2 [17]. Shapira et al reported that miR-625-3p concentration was elevated in plasma of patients with ovarian cancer and might serve as a biomarker to predict prognosis [18]. Our study showed that miR-625-3p promoted proliferation in ovarian high-grade serous carcinoma. These findings suggested that miR-625-3p plays important roles in high-grade ovarian serous carcinoma.

Cisplatin is used as a chemotherapy agent in variety of solid cancers including ovarian cancer. Resistance to cisplain-based chemotherapy is the major issue in patients with ovarian cancer. Different signaling pathways involve in development of cisplatin resistance including DNA damage repair, cell growth pathways and apoptotic pathways [19]. MicroRNAs have been demonstrated to involve in regulation of sensitivity of cisplatin in different neoplasms. For example, miR-34a could improve cisplatin sensitivity in non-small cell lung carcinoma [20] and miR-98-5p regulated Dicer1 axis and mediated cisplatin resistance in ovarian cancer [21]. We found miR625-3p overexpression improved growth of ovarian cancer cells with cisplatin treatment, however, low expression of miR-625-3p inhibited growth of ovarian cancer cells with cisplatin treatment. These findings suggested miR-625-3p regulated sensitivity of cisplatin in ovarian cancer cells. Furthermore, we showed miR-625-3p regulated BAX and bcl-2 expression in the presentence of cisplatin. These results indicated miR-625-3p might involve in cisplatin based chemotherapy resistance by regulating apoptosis pathway. We further showed miR-625-3p decreased PTEN expression in ovarian cancer cells by directly binding to 3’-UTR of PTEN. PTEN involves in development and progression in many neoplasms as tumor suppressor. Down-regulation of PTEN promotes cancer cell proliferation and inhibits apoptosis by regulating PI2K/AKT signaling.

Recent studies have shown that microRNAs involve in development of ovarian cancer by regulating PTEN signaling. Lou et al showed that miR-21 improved growth, migration and invasion in epithelial ovarian cancer cells by inhibiting PTEN expression [22]. Yang et al found that miR-214 and miR-150 were overexpressed in ovarian cancer cells and inhibited PTEN expression [23]. Li et al reported overexpression of miR-205 was associated with ovarian cancer progression by regulating both PTEN and SMAD4 signaling pathways [24]. Some studies also demonstrated that microRNAs involve in chemotherapy sensitivity in ovarian cancer by targeting PTEN signaling. Xiang et al reported miR-186 mediated cisplatin resistance in ovarian cancer cells by regulating PIK3R3 and PTEN signaling pathway [25]. Shi et al found miR-205-5p regulated cisplatin sensitivity by inhibiting PTEN expression in ovarian cancer cells [26].

In our study, PTEN expression was decreased in both ovarian cancer cell lines and human ovarian cancer specimen. These results indicated that miR-625-3p involved in ovarian proliferation via targeting PTEN signaling pathway.

In conclusion, our study suggested miR-625-3p might regulate growth and cisplatin based chemotherapy resistance in ovarian cancer by directly regulating PTEN signaling pathway. These findings indicate miR-625-3p could serve as a promising biomarker in ovarian cancer.

## Data Availability

The datasets used and/or analysed during the current study available from the corresponding author on reasonable request.

## Abbreviations

HGSOC: High-grade serous ovarian cancer
DMEM: Dulbecco’s Modified Eagle’s Medium
qRT-PCR: Quantitative Real-Time PCR
FFPE: Formalin-fixed paraffin-embedded
